# Telestroke activity across Europe; The results of a European Stroke Organization survey

**DOI:** 10.3389/fstro.2023.1282209

**Published:** 2024-03-25

**Authors:** Daniel J. Ryan, Peter Mueller-Barna, Rascha Von Martial, Francesco Corea, Bojana Zvan, Zeljko Zivanovic, Jesicaa Barlinn, Milena Krasinska-Chavez, Andrey Alasheev

**Affiliations:** ^1^Age-related Healthcare Department, Tallaght University Hospital, Tallaght, Dublin, Ireland; ^2^Department of Neurology and Neurological Intensive Care, Munchen Klinik Harlaching, Munich, Germany; ^3^Department of Neurology, TEMPiS Telestroke Center, Academic Teaching Hospital of the Ludwig Maximilians University, Munich, Germany; ^4^Stroke and Neurology Units, San Giovanni Battista Hospital, Turin, Italy; ^5^Neurology Clinic, National Center Telekap, University Medical Center Ljubjana, Lubjana, Slovenia; ^6^Faculty of Medicine, University of Novi Sad, Novi Sad, Serbia; ^7^Department of Neurology, Clinical Centre of Vojvodina, Novi Sad, Serbia; ^8^Department of Neurology, Technische Universitat Dresden, Dresden, Germany; ^9^NHS England, London, United Kingdom; ^10^Regional Stroke Center, Sverdlovsk Regional Clinical Hospital No. 1, Yekaterinburg, Russia

**Keywords:** telemedicine, telestroke, Europe, survey, thrombolysis

## Abstract

**Introduction:**

Telestroke care is likely not inferior to face-to-face care in acute stroke management while it also provides rural sites with access to specialist expertise. However, little is known about the distribution and activity of telestroke networks across Europe. Consequently, the European Stroke Organization (ESO) Telestroke Committee aimed to address this through an online questionnaire.

**Methods:**

The questionnaire was developed through an unstructured consensus process, ratified by the ESO Executive Committee, and emailed to ESO members.

**Results:**

Of 2,147 ESO members contacted, complete data sets were submitted on 25 networks from 10 countries. Among the 25 networks, the mean number of hubs per network was 1.6 (*SD* 1.2), and the mean number of spokes was 9 (*SD* 6.7), with considerable variability observed (range 2–24 spokes/network). All sites used audiovisual communication. The mean telemedicine consultations per year per site was 197 (*SD* 164). The primary reason for consultation was “diagnostic and triage purposes” in all but one network. The median number of strokes per site was 175 (interquartile range 192), and the mean intervention rate was 12.3% (*SD* 10; thrombolysis or thrombectomy).

**Conclusion:**

At 25 networks, this survey probably underrepresents telestroke activity across Europe, yet it is still the first study to provide a continent-wide geographical footprint and report on activity within the networks. There was considerable variability in network size and activity. Spoke sites reported an acceptable intervention rate of 12.3%. This percentage compares favorably with national data from European countries and suggests telestroke care supports reasonable intervention rates.

## Introduction

Telestroke has, for many years, provided an alternative to face-to-face treatment in hyper-acute stroke care (Bladin and Cadilhac, 2014). Randomized control trials, observational studies, and meta-analyses have compared telestroke care to face-to-face consultations and have consistently concluded it is likely not inferior to face-to-face acute care and results in similar adverse events (Meyer et al., 2008; Baratloo et al., 2018; Wysocki et al., 2019). Moreover, telestroke confers the obvious advantage of decentralizing acute care, possibly benefiting onset-to-treatment times (Hubert et al., 2016).

Telestroke's primary benefit lies in its ability to provide rural populations with 24/7 access to otherwise unavailable stroke-trained specialists (Kazley et al., 2012; Wu et al., 2014). One-quarter of European citizens live in rural areas, and disparities in accessing specialized stroke care probably contribute to suboptimal thrombolysis rates in regions within Europe [Europa, (n.d.)]. Establishing telestroke networks significantly enhances subsequent intervention rates and thus has been recommended by Angels initiatives (Müller-Barna et al., 2014; Wilcock et al., 2021)^1^. To date, local reimbursement and the availability of technology have proven to be barriers to telestroke expansion; however, the changing attitudes related to the pandemic at the European policy level and more ubiquitous use of telecommunication software may provide a favorable framework for expansion (Busti et al., 2021).

Much is unknown about the distribution and activity of telestroke networks across Europe. A German publication on network activity reports that networks are large, with median hubs being 1.5 hubs and median spokes being 9. All appear to practice a hub-and-spoke model of care and a drip-and-ship model (Barlinn et al., 2021). A French meta-analysis of telestroke care reports that all sites practiced a hub-and-spoke model (2–20 spokes per network) and a drip-and-ship model (Ohannessian et al., 2020). A publication from Cambridge in the United Kingdom has reported on an organically evolved parallel or “horizontal” model without a clear hub site (Agarwal et al., 2014). Sporadic publications on activity across Europe have provided a patchwork impression of networks that may have evolved through iterative growth or local drivers rather than a systematic approach (Barlinn et al., 2021; Busti et al., 2021). However, sporadic publications likely do not represent Europe-wide telestroke activity. Consequently, the Telestroke Committee of the European Stroke Organization (ESO) followed its telestroke guideline publication with a telestroke survey of all ESO members (Hubert et al., 2018). The aim was to map areas of telemedicine activity, ascertain the nature of configuration and activity, and provide network examples for those wishing to evolve a new service.

## Methods

The ESO Telestroke Committee developed an online questionnaire over two online sessions, utilizing an unstructured consensus process. Once finalized by the committee, the draft questionnaire was reviewed by the ESO Executive Committee. The survey requested information regarding the model and size of the networks, whether audio and visual communication took place, teleconsultation activity, and intervention rates in the networks (see the Supplementary material for the data from the full survey).

Once ratified by the committee, it was promoted and disseminated by directly emailing all ESO members and with an ESO social media launch. ESO members were requested to complete an online form (https://survey.lamapoll.de/ESO_Telestroke_Survey_2021). In addition, the committee wrote to the national stroke leads within Europe. The survey was launched on February 7, 2021, and remained accessible for 1 year. Incomplete data submissions were individually followed up by the committee members. Ethical approval was not sought for the present study because the study participants were ESO members rather than patients and because consent was implied through response to our email contact.

Statistical analysis was performed using Stata 14. Continuous variables were presented as means with corresponding standard deviations (*SD*) or medians with corresponding interquartile ranges (IQR). Dichotomous variables were presented with their absolute numbers and percentages. In the case of continuous variables, baseline characteristics and outcomes between two groups were compared using a *t*-test if the variables had a normal distribution or the Mann–Whitney *U*-test if not. The Shapiro–Wilk test was used to assess the normality of distribution.

## Results

**Figure 1:**
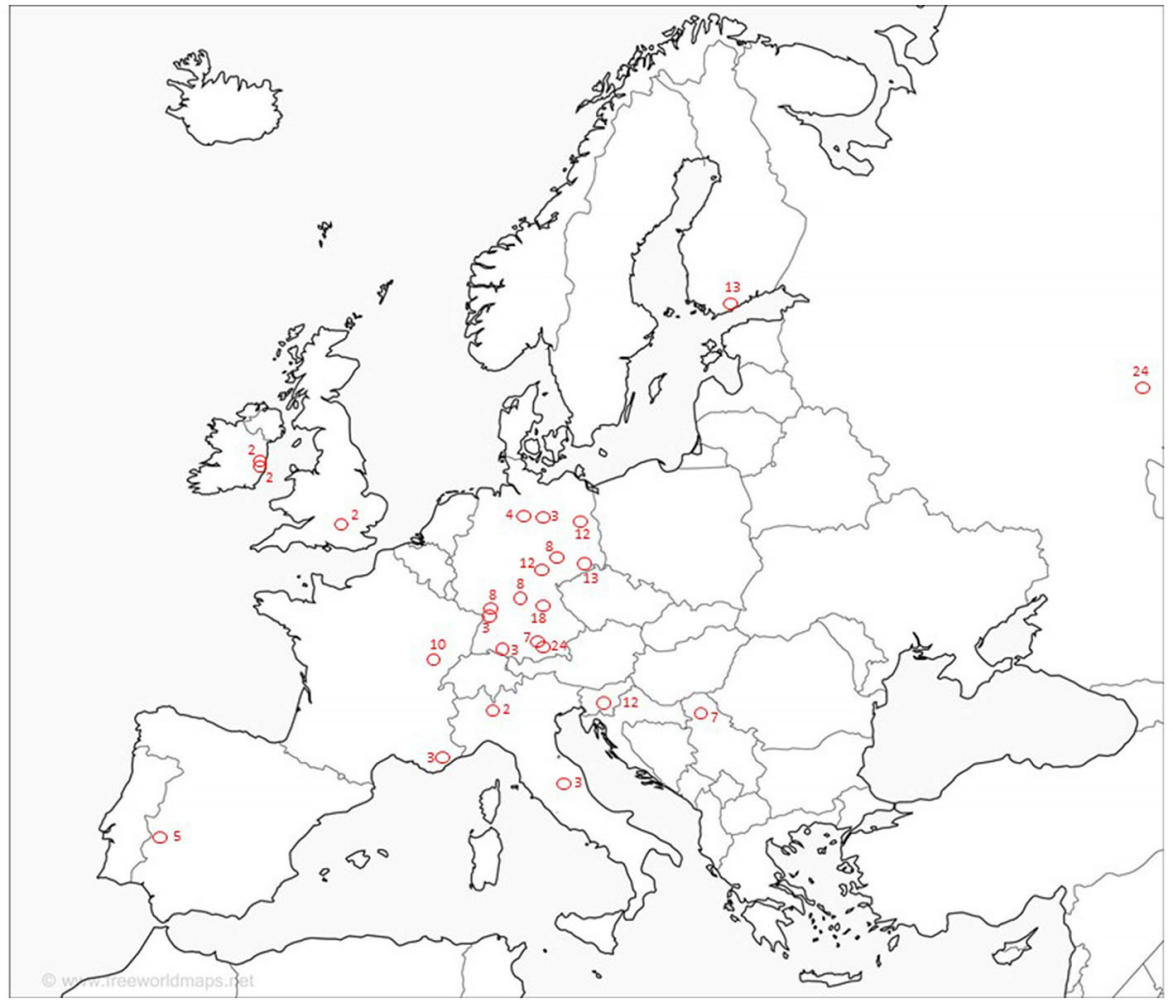
Location of telemedicine networks and number of spokes per network.

The survey was emailed to all 2,147 ESO members. The email was opened by 1,122 members, and the ESO committee received 34 submissions; 8 were incomplete, and 1 was a duplication. After follow-up, the ESO Telestroke Committee received 25 complete submissions. Networks were described in 10 countries; Germany accounted for 13 networks; Italy, 2; France, 2; and Ireland, 2, as well as 1 each from Spain, Finland, Russia, Slovenia, Serbia, and the United Kingdom (see Figure 1). Four incomplete data sets were also acquired from Spain, France, Italy, and Ukraine and were not included in the analysis. All completed responses described a hub-and-spoke model, with the hub in an urban area; the mean hub population was 864,236 (*SD* 1.8 million).

Hub–spoke activity and characteristics are presented in Table 1. The mean number of hubs in a network was 1.6 (*SD* 1.2, range 1–6), while the mean number of spokes in a network was 9 (*SD* 6.7, range 2–24). All sites used both audio and visual communication systems. The median distance from the hub site to the spoke site was 70 km (IQR 55 km). There was considerable variability in hub–spoke distance. Germany, for example, had a median distance from hub to spoke of 46 km (IQR 32), and Italy was similar, with a median distance of 45 km (IQR 12). Finland, by comparison, had a median distance from hub to spoke of 330 km (IQR 382).

**Table 1:** Characteristics of telemedicine networks in Europe.

**Description**	***N***
Telemedicine networks	25
Number of countries	10
Number of hubs per network, mean (range)	1.6 (1–6)
Number of spokes per network, mean (range)	9 (2–24)
Distance from hub site to spoke site, km median (IQR)	70 (55)
Telemedicine consultations per year, mean (*SD*) [range]	2310 (*SD*)
	[25–10,240]
Telemedicine consultations per spoke per year, mean (*SD*) [range]	197 (164)
	[20–468]
Strokes per year in all spoke sites in the network, median (IQR)	2107 (2,049)
Strokes per year per site, median (IQR)	175 (192)
Interventions per year in all sites in the network, median (IQR)	255 (210)
Interventions per year per site, median (IQR)	15 (24)
Intervention rate, median % (IQR)	9.6 (15)
**Primary use of telemedicine service**	
Emergency room consultation, %	24 (96)
TIA clinic, %	1 (4)
Audio-visual both sides, %	25 (100)
**Maintenance of a register**	
Single register for all sites, %	14 (56)
Spokes maintain their own register, %	5 (20)
No register, %	7 (28)

The mean number of telemedicine consultations per year per network was 2,310 (*SD* 2,721, range 25–10,240) or 197 per spoke (*SD* 164, range 20–468). The primary reason for telemedicine consultations was an emergency room consultation for “diagnostic and triage purposes” in all but one site. One network in the United Kingdom used telemedicine for a transient ishcaemic attack (TIA) service. The median number of strokes per network per year was 2,107 (IQR 2,049, range 100–22,430). The median number of interventions per network per year (denoted “reperfusion therapies” in the survey) was 255 (IQR 210, range 10–1,320). Representing this activity per spoke site generates a median number of strokes per spoke site per year of 175 (IQR 192) and a median number of interventions per spoke site per year of 15 (IQR 24). This activity equates to a median intervention rate of 9.6% (IQR 15) and a mean intervention rate of 12.3% (*SD* 10).

**Figure 2:**
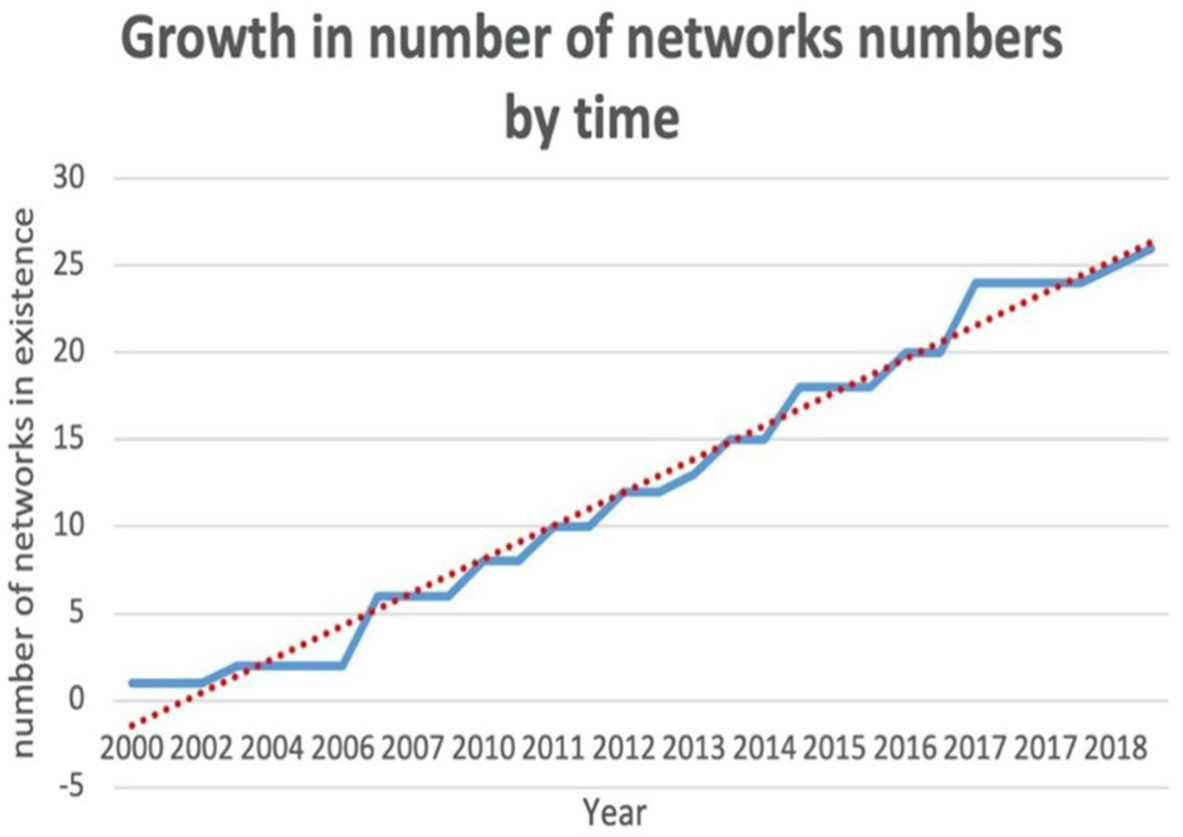
Growth in the number of telemedicine networks according to reported year they commenced.

**Figure 3:**
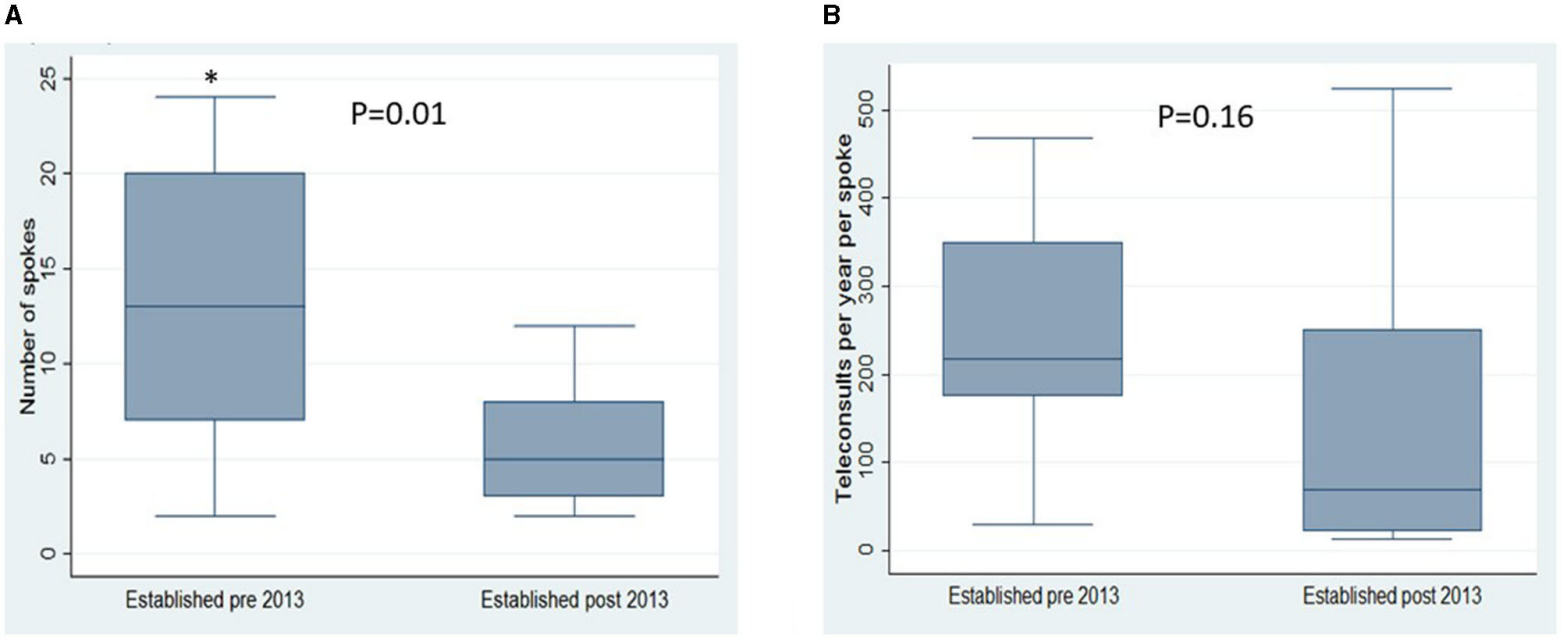
Comparison of earlier established networks with that of later established networks (groups dichotomised according to the median year of network commencement, 2013). Compared according to **(A)** Number of spokes in the network and **(B)** Teleconsultations per site per spoke. ^*^p < 0.05.

Telemedicine networks have observed a linear growth in Europe over the last 20 years (see Figure 2). The median year in which a network commenced was 2013. We dichotomized networks into those established before 2013 and those established after 2013. We observed that networks established before 2013 had significantly more spoke sites than those established after 2013; the mean number of spokes was 12.9 (*SD* 7.8) vs. 5.8 (*SD* 3.6), *p* = 0.01. We also observed a non-significant trend toward more frequent teleconsultations per year per spoke site in networks established before 2013 than those after 2013; teleconsultations per year per spoke site were 216 (IQR 176) in those established before 2013 vs. 68 teleconsultations per year per spoke site in those established after 2013 (IQR 230), *p* = 0.16 (see Figure 3).

## Discussion

This ESO-supported Europe-wide telestroke survey describes a linear growth in the number of telestroke networks across Europe. These networks are usually quite far-reaching, with a mean of 9 spokes and 1.5 hubs per network or, alternatively, 6 spokes per hub. This network configuration mirrors a U.S. survey that reported a mean of 7.6 spokes per hub (Silva et al., 2012). Across Europe, networks vary considerably in size, ranging from 2 spokes to 24 spokes per network. The distance from a hub-to-spoke site varied from 3 km (Germany) to 868 km (Finland). Activity also varies considerably, ranging from 20 tele-consultations per site per year to 468 tele-consultations per site per year.

Networks that developed more recently (after 2013) were smaller and exhibited a trend toward fewer teleconsultations/year/site. Earlier established telemedicine networks probably continue to grow after inception, or early adaption possibly occurred in places of greater need. Evidence for the former is supported by the aforementioned U.S. telestroke survey that observed a significant growth in the number of spokes per hub during the monitored period between 2007 and 2009, 3.78 vs. 7.60, *p* = 0.05 (Agarwal et al., 2014). So it is likely that the intra-network growth is iterative.

All networks but one use the service to support emergency department consultations for acute stroke triage and assessment. All networks report using audio and visual solutions for communication. Given the pre-COVID-19 inception date, this suggests the use of telestroke-specific software, but the survey did not request this information.

The reported overall median intervention rate among the spoke sites was 9.6% (mean 12.8%), which is adequate, given that the majority of interventions will have taken place in smaller, non-university hospitals. This compares favorably with publications from other European sites, albeit assessed at earlier time points [Stroke Europe, (n.d.)].

The survey has several limitations. Most notably, our capture rate was probably inadequate. A 2009 literature review by BM Demaershcalk et al. reported on telestroke-specific activity and identified five telestroke-specific networks in Europe at the time, which correlates with the findings of this survey for that time period (see Figure 2) (Demaerschalk et al., 2009).

However, a recently published German paper identified 22 telemedicine networks in Germany alone (Barlinn et al., 2021). Of these 22 networks, 13 responded to our survey (60% response rate). A French meta-analysis reported seven hub-and-spoke networks in France, of which three responded to our survey (one incomplete data set) (Ohannessian et al., 2020). Consequently, it is reasonable to conclude that this survey is not entirely representative of Europe-wide telemedicine activity. Moreover, in this survey, we did not explore the technical elements or quality indicators in the networks as this is the intention of future ESO telestroke surveys. The intent of this survey was to identify the location of networks, report on the geographic configuration of spokes, and describe basic activity, with the intention of forming a footprint for future mapping of activity and growth.

Limitations notwithstanding, we report the first Europe-wide description of telemedicine activity derived from non-direct email communication with more than 2,000 ESO members and follow-up communication with national stroke leads. The ongoing growth of networks is encouraging, and the variety in size and activity should encourage those aspiring for small collaborations with iterative growth in the likely observed model. Intervention rates among networks are greater than expected for what are predominantly smaller, more rural hospital sites supported by urban hub sites.

This telestroke map, if accurate, points toward clear geographical “black spots” across Europe, and clinicians can use these data to influence national policy toward a more favorable regulatory and incentive-based environment, especially in a post-COVID-19 videoconferencing era.

## Data availability statement

The raw data supporting the conclusions of this article will be made available by the authors, without undue reservation.

## Author contributions

DR: Data curation, Formal analysis, Funding acquisition, Investigation, Methodology, Project administration, Resources, Software, Supervision, Validation, Visualization, Writing—original draft, Writing—review & editing. PM-B: Conceptualization, Data curation, Investigation, Methodology, Project administration, Resources, Software, Supervision, Validation, Writing—review & editing. RV: Conceptualization, Formal analysis, Investigation, Methodology, Project administration, Supervision, Visualization, Writing—review & editing. FC: Conceptualization, Data curation, Formal analysis, Investigation, Methodology, Project administration, Supervision, Visualization, Writing—review & editing. BZ: Conceptualization, Investigation, Methodology, Project administration, Supervision, Visualization, Writing—review & editing. ZZ: Conceptualization, Investigation, Methodology, Writing—review & editing. JB: Conceptualization, Data curation, Formal analysis, Methodology, Software, Visualization, Writing—review & editing. MK-C: Conceptualization, Investigation, Methodology, Project administration, Software, Visualization, Writing—review & editing. AA: Conceptualization, Formal analysis, Methodology, Project administration, Software, Visualization, Writing—review & editing, Data curation, Investigation, Resources, Supervision, Validation, Writing—original draft.

## Footnotes

^1^ www.Angels-initiative.com
