# Supplementary material for: Telestroke activity across Europe; The results of a European Stroke Organization survey
Source: Front Stroke. 2024 Mar 25;2:1282209. doi: 10.3389/fstro.2023.1282209 (PMC12802621; doi:10.3389/fstro.2023.1282209)
Supplement: Supplementary file 1 [file Data_Sheet_1.pdf]

Survey emailed to ESO members

Domain	Question
HQ	What is the name of your telestroke headquater?
HQ	In which city is the headquater based?
HQ	In which country is the headquater based?
HQ	What is the name of your telestroke network?
HQ	In which year was your telestroke network established?
HQ	details of the primary contact person of your network?
HQ	.. lastname …
HQ	… and email adress.
Network	What is the organizational model of your telestroke network?<br>Please select one from the list.
Network	If you choose "other", please describe your network model.
Network	How many hubs does your network have?
Network	What is the name of your telestroke hub?
Network	In which city is the hub based?
Network	In which country is the hub based? Please select one from the list.
Network	Please provide detailed list of the other hubs. Including name, city, country.
Network	How many spokes does your network have?
Network	Please provide detailed list of the spokes. Including name, city, country.
Stroke_care	Please provide the number of stroke patients admitted to your spoke hospitals in 2019.
Stroke_care	Please provide the number of stroke patients with reperfusion in 2019?
Stroke_care	What is the overall number of teleconsultations in 2019?
Stroke_care	What are the main indications for teleconsultation in your network?<br>multiple selections possible
Stroke_care	If you choose "other", please describe here.
Comunication	What is the type of communication used in your network?<br>Multiple selections possible
Register	What kind of registry does your network provide?
